# Bovicin HJ50-Like Lantibiotics, a Novel Subgroup of Lantibiotics Featured by an Indispensable Disulfide Bridge

**DOI:** 10.1371/journal.pone.0097121

**Published:** 2014-05-12

**Authors:** Jian Wang, Hongchu Ma, Xiaoxuan Ge, Jie Zhang, Kunling Teng, Zhizeng Sun, Jin Zhong

**Affiliations:** 1 State Key Laboratory of Microbial Resources, Institute of Microbiology, Chinese Academy of Sciences, Beijing, PR China; 2 University of Chinese Academy of Sciences, Beijing, PR China; La Trobe University, Australia

## Abstract

Lantibiotics are ribosomally-synthesized and posttranslationally modified peptides with potent antimicrobial activities. Discovery of novel lantibiotics has been greatly accelerated with the soaring release of genomic information of microorganisms. As a unique class II lantibiotic, bovicin HJ50 is produced by *Streptococcus bovis* HJ50 and contains one rare disulfide bridge. By using its precursor BovA as a drive sequence, 16 BovA-like peptides were revealed in a wide variety of species. From them, three representative novel *lan* loci from *Clostridium perfringens* D str. JGS1721, *Bacillus cereus* As 1.348 and *B. thuringiensis* As 1.013 were identified by PCR screening. The corresponding mature lantibiotics designated perecin, cerecin and thuricin were obtained and structurally elucidated to be bovicin HJ50-like lantibiotics especially by containing a conserved disulfide bridge. The disulfide bridge was substantiated to be essential for the function of bovicin HJ50-like lantibiotics as its disruption eliminated their antimicrobial activities. Further analysis indicated that the disulfide bridge played a crucial role in maintaining the hydrophobicity of bovicin HJ50, which might facilitate it to exert antimicrobial function. This study unveiled a novel subgroup of disulfide-containing lantibiotics from bacteria of different niches and further demonstrated the indispensable role of disulfide bridge in these novel bovicin HJ50-like lantibiotics.

## Introduction

Disulfide bridges especially intramolecular disulfide linkages are prevalent in antimicrobial peptides and have been acknowledged to play important roles in biological function either by dictating the complex structural conformation or maintaining stability. The best-studied disulfide-containing antimicrobial peptides are host defense peptides that comprise multiple disulfide connectivities, which are widely distributed among human, animals, plants and even fungi [1], [2]. Bacteriocins are ribosomally-synthesized antimicrobial peptides produced by a wide range of bacteria from lactic acid bacteria to actinobacteria and are divided into lantibiotics and nonlantibiotics [3]. Disulfide bridge is extensively found in nonlantibiotics especially pediocin-like bacteriocins, which contain a crucially conserved disulfide bridge at their N termini while in some cases contain an additional one at C termini [4]. However, disulfide bridge is rarely found in lantibiotics in spite of a high proportion of Cys residues included.

As a large member of bacteriocins, lantibiotics are posttranslationally modified peptides mainly containing lanthioine (Lan) and methyllanthioine (MeLan) residues [5], [6]. These unusual residues are introduced by lanthionine synthetases, based on which lantibiotics are classified into four classes (I to IV) [6]. Class I lantibiotics are dehydrated by a dedicated dehydratase LanB and cyclized by a cyclase LanC. Class II lantibiotics are dehydrated and cyclized by one bifunctional LanM. Class III and Class IV lantibiotics are catalyzed by a tridomain protein LabKC and LanL respectively, which differ in their C-terminal cyclase domain. A recently discovered novel labionin (Lab) is generated by a subset of class III enzymes [7]. Meanwhile, other diverse enzymatically catalyzed modifications have also been revealed in lantibiotics, leading to special residues such as S-aminovinyl-D-cysteine (AviCys), 2-oxobutyryl (OBu) and lysinoalanine, halogenated tryptophan and hydroxylated aspartic acid [5], [8], [9]. Interestingly, although containing a high percentage of Cys residues, lantibiotics seldom contain disulfide connections as Cys residues are in most cases enzymatically cross-linked with dehydrated Ser or Thr to form thioether or carbacyclic linkages. To date, disulfide bridge has only been revealed in few lantibiotics like the α subunit of two-component lantibiotics including haloduracin α [10], plantaricin Wα [11], enterocin Wα [12] and recently discovered unique class III lantibiotics labyrinthopeptin A1 and A2 [7]. However, the enzyme(s) involved in disulfide bridge formation was not identified in gene clusters responsible for lantibiotic synthesis. The function of these disulfide bridges have not been fully investigated and were indicated to be of minor importance to biological activities [7], [10], [11].

Bovicin HJ50 is a 34-amino acid class II lantibiotic produced by *Streptococcus bovis* HJ50 [13]. It was firstly grouped into lacticin 481 subgroup as its topology is reminiscent of lacticin 481 with an N-terminal linear and C-terminal globular structure [14]. However, bovicin HJ50 distinguishes from other lacticin 481-like lantibiotics by containing a unique disulfide bridge, and its NMR structure has been recently elucidated (PDB ID: 2M8V). This disulfide bridge was demonstrated to be critical for the antimicrobial activity of bovicin HJ50 [14]. Thermophilin 1277 from *S. thermophilus* and recently found macedovicin from *S. macedonicus* turned out to be identical to bovicin HJ50 [15], [16]. Thus, bovicin HJ50 seems to be a unique disulfide-containing lantibiotic.

Hereby, via genomic mining in NCBI database using BovA as a drive sequence, a series of putative bovicin HJ50-like lantibiotic gene clusters were revealed in a broad range of species like *Streptococcus suis, Enterococcus columbae*, *Clostridium perfringens, Bacillus cereus* and *B. thuringiensis*. Recently, we have biosynthesized one bovicin HJ50-like lantibiotic suicin from a remnant *sui* locus via a semi-*in vitro* biosynthesis (SIVB) system by reconstitution of the disrupted modification gene *suiM* in *Escherichia coli* and subsequent *in vitro* digestion with peptidase domain of SuiT [14], [17]. In this study, three other bovicin HJ50-like lantibiotics named perecin, cerecin and thuricin were obtained and characterized, which were all novel lantibotics from separate species. They are hereby assigned into a novel bovicin HJ50 subgroup of disulfide-containing lantibiotics. In all these bovicin HJ50-like lantibiotics, disulfide bridge was proved to be crucial to their bioactivity, which was unprecedented in other lantibiotics.

## Results

### Revealing of bovicin HJ50-like lantibiotic gene clusters by genomic mining and PCR screening

By using bovicin HJ50 precursor BovA as a drive sequence, more than 16 BovA-like proteins had been revealed from publicly available genomes of streptococci, enterococci, clostridium and bacilli in NCBI database (**Table S1**). These BovA-like peptides are small in size (<55aa) and display more than 36% identities with BovA. They all contain a typical class II lantibiotic leader peptide with a GG motif for cleavage to release mature lantibiotics and the putative core peptides are conserved in residual positions of two dehydratable Thr and four ring-forming Cys as bovicin HJ50 (**Figure S1**). Especially, the TLTKDCP motif that constitutes the ring A in bovicin HJ50, is almost identical in these BovA-like peptides. Thus, these hits are speculated to be putative bovicin HJ50-like lantibiotic precursors. Precursors of thermophilin 1277 (ThmA) and recently discovered macedovicin (MdvA) are identical to BovA. Another BovA-identical precursor ColA, was revealed in *E. columbae* PLCH2. SuiA is from a remnant *sui* locus that has recently been unveiled in pathogenic *S. suis* serotype 2, and the corresponding suicin has been produced via a semi-*in vitro* biosynthesis system [17]. Other precursors are from strains of pathogenic *C. perfringens* and opportunistic pathogens *B. cereus* and *B. thuringiensis*.

**Figure 1:**
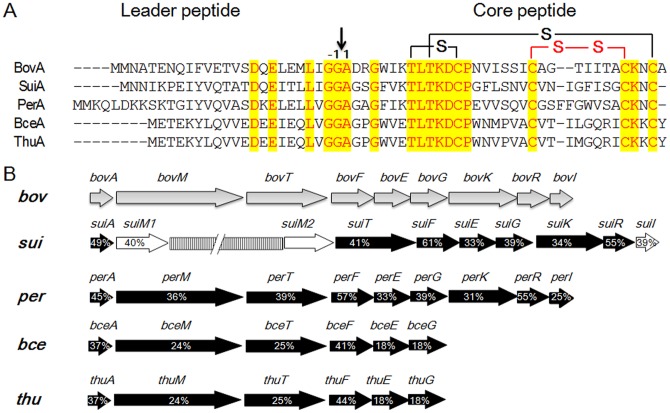
Revealing of bovicin HJ50-like lantibiotic gene clusters. (A) Sequence analysis of precursor peptides of BovA-like peptides including BovA, SuiA, PerA, BceA and ThuA. The residues in red of yellow background indicated identical amino acids in all aligned precursors. The thioether bridges (black) or disulfide bridge (red) illustrated the ring topology of bovicin HJ50. The vertical arrow indicated the processing site (between Gly-1 and Ala1) for BovT to release mature bovicin HJ50. (B) Bovicin HJ50-like lantibiotic gene clusters. Percentages in horizontal arrows represented the identities of the lantibiotic biosynthesis-related proteins with that of *bov* locus.

BovA-like peptides (BceAs and ThuAs) are abundant in bacilli strains, which are our targets for PCR mining. Primers were designed according to a highly identical region of BceAs and ThuAs namely METEKYL…ICKKC. PCR screening was performed over five *B. cereus* (As 1.1846, 1.260, 1.348, 1.352 and 1.447) and three *B. thuringiensis* (As 1.1013, 1.1014 and 1.913) strains. The *bceA* was detected in one *B. cereus* strain As 1.348 and *thuA* was detected in two *B. thuringiensis* strains As 1.1013 and As 1.1014. Protein sequencing analysis revealed that ThuAs from *B. thuringiensis* As 1.1013 and As 1.1014 were identical while different with BceA from *B. cereus* As 1.348 by only two residues (**Figure 1A**). BceA showed high identity with BceA5 (EJR27159) from *B. cereus* VD048 and ThuA showed high identity with ThuA3 (AGG04873) from plasmid of *B. thuringiensis* str. IS5056. With primers designed according to the analogical loci, the complete lantibiotic gene clusters were subsequently amplified and sequenced.

The gene cluster from *B. cereus* As 1.348 was designated *bce* locus (accession number: KJ504103) and the one in *B*. *thuringiensis* As 1.013 was termed *thu* locus (accession number: KJ504104). The *thu* locus from *B*. *thuringiensis* As 1.014 was identical to that from *B*. *thuringiensis* As 1.013. The complete *bce* locus encompasses six genes, including the structural gene *bceA*, one modification gene *bceM*, one transporter gene *bceT* and three immunity-related genes *bceFEG* (**Figure 1B**). BceA showed 37% identity with BovA and BceM showed 24% identity with BovM, indicating the possible role of BceM in modification of BceA. The *bceT* might encode an ABC transporter whose putative product shows 25% identity with BovT and contains an N-terminal C39 peptidase domain as BovT. BceF, E and G have 41%, 18% and 18% identity with BovF, E and G respectively, which encode membrane-anchored ABC transporters involved in self-immunity. The *thu* locus displays 96% identity with *bce* in nucleotide sequence, indicating the same function as *bce*. The PerA (EDT72694) was from a genome-sequenced pathogenic strain *C*. *perfringens* D str. JGS1721, and the corresponding gene cluster was designated *per* locus (CJD_0433-CJD_0441). The *per* locus was more analogous to *bov* by containing a structural gene (*perA*), a modification gene (*perM*), an ABC transporter gene (*perT*), a two-component transduction system (*perKR*) and self-immunity related genes (*perFEG* and *perI*). Moreover, the biosynthesis-related genes showed high identity with their counterpart in *bov* locus (**Figure 1B**). These putative novel *lan* loci especially *per* locus might be involved in lantibiotic production, whereas these strains have not been reported to produce any lantibiotics.

### Semi-*in vitro* biosynthesis (SIVB) and structural dissection of bovicin HJ50-like lantibiotics

**Figure 2:**
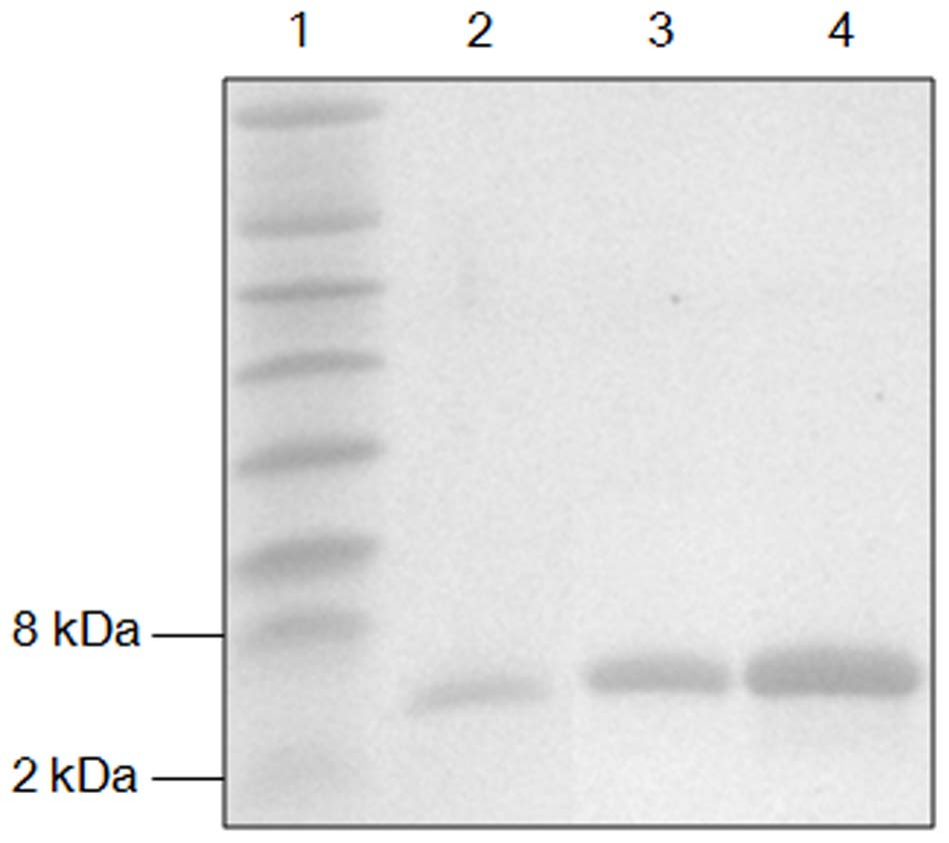
Tricine-SDS-PAGE of mature bovicin HJ50-like lantibiotics. Lane 1, protein marker; lane 2, perecin; lane 3, cerecin; lane 4, thuricin.

**Figure 3:**
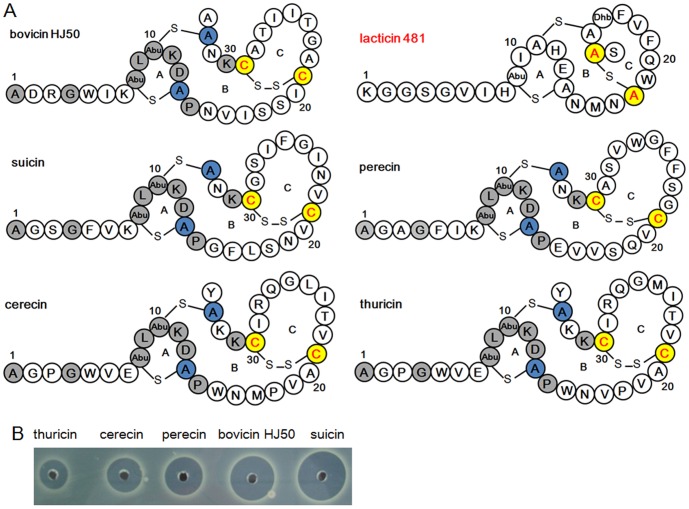
Structure and antimicrobial activity of bovicin HJ50-like lantibiotics. (A) Comparison of structure of bovicin HJ50-like lantibiotics with lacticin 481. Gray residues indicated identical amino acids and the Cys residues in red of yellow background indicated disulfide-forming Cys in bovicin HJ50, suicin, perecin, cerecin and thuricin. (B) Comparison of antimicrobial activity of bovicin HJ50-like lantibiotics. In each hole, 25 µL of 10 µg/mL compounds were applied.

Semi-*in vitro* biosynthesis (SIVB) system consisting of *in vivo* modification and *in vitro* processing has been demonstrated to be an efficient way to produce lantibiotics [14], [18]. Bovicin HJ50 and suicin have been biosynthesized previously with this strategy [14], [17]. Therefore, to synthesize putative lantibiotics using SIVB, *bceAM*, *thuAM* and *perAM* were amplified from genome of respective strains and were then cloned onto pET28a to obtain pET28a-*bceAM*, pET28a-*thuAM* and pET28a-*perAM*, respectively, which were then transformed into *E. coli* BL21(DE3) for protein expression. Modified precursor peptides His_6_-mBceA, His_6_-mThuA and His_6_-mPerA were purified by ion metal affinity chromatography (IMAC). After digestion by His_6_-BovT_150_, the N-terminal C39 peptidase domain (150aa) of bovicin HJ50 transporter BovT, mature thuricin and perecin were purified by reverse phase high performance liquid chromatograph (RP HPLC) with C_18_ column and determined by Tricine-SDS-PAGE (**Figure 2**). MALDI-TOF MS analysis showed the [M+H]^+^ of perecin and thuricin as 3514.36 Da and 3728.76 Da, which were 38.31 Da and 38.07 Da decrease compared with separate calculated mass, respectively (**Table 1** and **Figure S2**). However, cerecin was not modified. We attempted to delete the spacer region between *bceA* and *bceM* by linking the two genes with only *Bam*HI site in between. BceA was then modified by BceM *in vivo* and the mature cerecin was produced. MS analysis showed [M+H]^+^ of cerecin as 3742.46 Da, which was 38.39 Da decrease in comparison with theoretical molecular weight (**Table 1**). Antimicrobial assay exhibited that all biosynthesized bovicin HJ50-like lantibiotics were active against indicator strain *Micrococcus flavus* NCIB8166 (**Figure 3B**). IC_50_ of bovicin HJ50-like lantibiotics were determined against sensitive indicator strain *M. flavus* NCIB8166, which was 0.195 µg/mL for suicin, 0.39 µg/mL for bovicin HJ50, 0.78 µg/mL for perecin, 1.625 µg/mL for cerecin and 3.125 µg/mL for thuricin, respectively (**Table 1**).

**Table 1:** MS analysis and IC_50_ determination of bovicin HJ50-like lantibiotics.

Lantibiotics	MW cal.^a^ (Da)	MW by MS (Da)	ΔMW(Da)	PTM^b^	IC_50_ c (µg/mL)
bovicin HJ50	3466.73	3428.65	38.08	2H_2_O+S-S	0.39
suicin	3379.63	3341.28	38.35	2H_2_O+S-S	0.195
perecin	3552.67	3514.36	38.31	2H_2_O+S-S	0.78
cerecin	3780.85	3742.46	38.39	2H_2_O+S-S	1.625
thuricin	3766.83	3728.76	38.07	2H_2_O+S-S	3.125

a Calculated molecular weight (MW) of protonated peptides.

b Post translational modification.

c The 50% inhibitory concentration.

Bovicin HJ50 and suicin had been structurally elucidated to be similar as an N-terminal linear and C-terminal globular structure [14], [17]. High similarity of core peptides implied the structural similarity of bovicin HJ50-like lantibiotics. Sequence analysis showed that residues of ring A are almost identical and the dehydratable Thr residues and the ring-forming Cys residues are conserved in bovicin HJ50-like lantibiotics. As anticipated, the approximate 38 Da decrease in mass indicated two dehydrations and one disulfide bridge in perecin, cerecin and thuricin (**Table 1**). To determine the ring topology, each Cys in perecin and cerecin was mutated to Ala (**Table S2**). C13A or C34A of perecin and C13A or C33A of cerecin were 38 Da decrease in mass while C21A or C31A of perecin and C21A or C30A of cerecin were 36 Da decrease. This indicated that Cys13 and Cys34 of perecin, as well as Cys13 and Cys33 of cerecin, were involved in thioether bridge formation, while Cys21-Cys31 in perecin and Cys21-Cys30 in cerecin formed disulfide linkage. This verified that perecin and cerecin are similar to bovicin HJ50 in structure (**Figure 3A**). Thuricin was supposed to be structurally similar to cerecin in that thuricin differed from cerecin only by two residues (Met17 and Leu25 in thuricin, Val17 and Met25 in cerecin). Thus, three novel disulfide-containing lantibiotics were obtained and were structurally assigned to be bovicin HJ50-like lantibiotics (**Figure 3A**).

### *In vitro* formation of disulfide bridge

**Figure 4:**
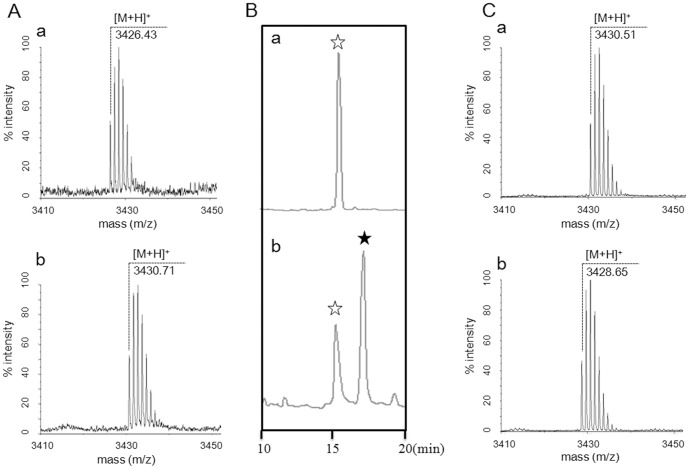
MS and HPLC analysis of bovicin HJ50 and its *in vitro* produced counterparts. (A) a, bovicin HJ50 produced by BovM *in vitro* in the absence of DTT; b, main product produced by BovM *in vitro* in the presence of DTT. (B) a, HPLC analysis of reduced bovicin HJ50; b, HPLC analysis of reduced bovicin HJ50 after exposed to air for 2 days. The white star indicated reduced bovicin HJ50 and the black star indicated the wild type bovicin HJ50. (C) MS analysis of reduced bovicin HJ50 (a) and wild type bovicin HJ50 (b).

BovM was expressed to modify BovA *in vitro* to determine if BovM introduced disulfide bridge into bovicin HJ50. His_6_-BovA was incubated with His_6_-BovM in the reaction buffer containing ATP and Mg^2+^. The modified products were processed by His_6_-BovT_150_ before MS analysis. When DTT was absent, a main product with molecular weight of 3426.43 Da was detected (**Figure 4A-a**). This was in good accordance with calculated molecular weight of BovA core peptide with two dehydrations and two disulfide bridge formations. The tandem MS analysis of this product confirmed that no thioether bridge was formed (**Figure S3**). However, when DTT was present in the reaction buffer, MS analysis exhibited a mass peak of 3430.71 Da (**Figure 4A-b**), which was 36.02 Da decrease compared with theoretical mass of BovA core peptide (3466.73 Da). This indicated that BovM did not contribute to disulfide bridge formation in bovicin HJ50. This also suggested that a reduced condition was in favor of BovM function in catalyzing thioether bridge formation.

When analyzed by RP HPLC with C_18_ column, bovicin HJ50 was eluted with a retention time of 17.5 min. Bovicin HJ50 was firstly reduced with reductive reagent TCEP (tris(2-carboxyethyl) phosphine) and the reduced bovicin HJ50 (bovicin HJ50 Red) was purified to homogeneity by RP HPLC with retention time of 15.7 min (**Figure 4B-a**). The lyophilizd bovicin HJ50 Red was dissolved in H_2_O and exposed to air for more than 2 days. The following RP HPLC analysis showed that another peak corresponding to wild type bovicin HJ50 showed up (**Figure 4B-b**). MS analysis confirmed the molecular weight of corresponding reduced bovicin HJ50 (**Figure 4C-a**) and wild type bovicin HJ50 (**Figure 4C-b**). This indicated that in native condition, the disulfide bridge is favored in bovicin HJ50.

### Impact of disulfide bridge breakage on antimicrobial activity

**Figure 5:**
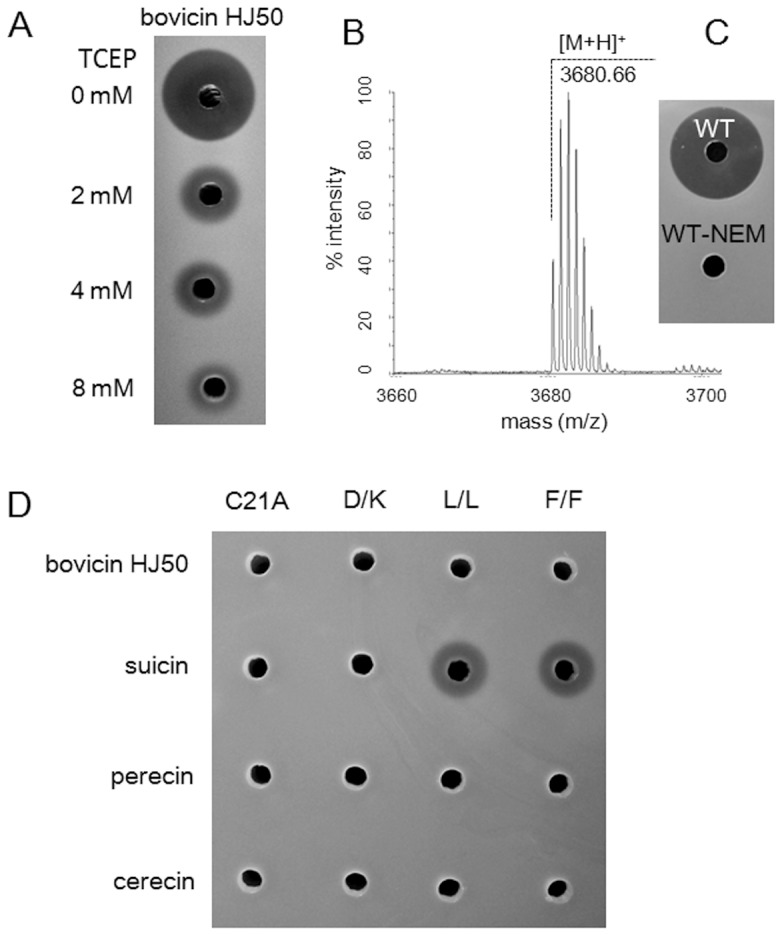
Mutation, substitution and alkylation of disulfide bridge in bovicin HJ50-like lantibiotics. (A) Antimicrobial activity of wild type bovicin HJ50 (25 µL, 5 µg/mL) when treated respectively with 0, 2, 4, 8 mM TCEP. (B) MS analysis of purified NEM-alkylated bovicin HJ50. (C) Antimicrobial activity of wild type bovicin HJ50 (WT) and alkylated bovicin HJ50 (WT-NEM). (D) Antimicrobial activity of disulfide-related mutants. D/K represents substitution of disulfide-forming Cys residues to Asp and Lys, e.g., bovicin HJ50 D/K means bovicin HJ50 mutant C21D/C29K. L/L or F/F is referred as the same condition as D/K. 25 µL peptide samples of 20 µg/mL were applied to each hole.

To determine if the conserved disulfide bridge is critical to the function of these bovicin HJ50-like lantibiotics, disulfide bridge was disrupted by reduction, alkylation or mutation, respectively. Bovicin HJ50 of 5 µg/mL was incubated with increasing concentration of TCEP from 2 mM to 8 mM for 2 h. Antimicrobial assay exhibited that reduced bovicin HJ50 displayed gradually decreased activity against indicator strain *M. flavus* NCIB8166 with the increasing concentration of TCEP (**Figure 5A**). For further confirmation, reduced bovicin HJ50 was alkylated by NEM (N-ethylmaleimide, 125 Da) which was reactive with free thiols. Purified fully alkylated bovicin HJ50 showed an [M+H]^+^ of 3860.66 Da (**Figure 5B**), which was 252.01 Da higher than the mass of bovicin HJ50 (3428.65 Da). This indicated the opening of disulfide bridge and addition of 2 NEM molecules in bovicin HJ50. Antimicrobial assay showed that the disulfide shielded bovicin HJ50 was inactive (**Figure 5C**). Further mutation of disulfide-forming Cys residues to Ala was performed. C21A mutants of bovicin HJ50, suicin, perecin and cerecin abolished their activity to inhibit sensitive indicator *M. flavus* NCIB8166 (**Figure 5D**). These results all underscored that disulfide bridge was critical to the bioactivity of bovicin HJ50-like lantibiotics.

### Substitution of disulfide bridge with salt bridge and hydrophobic interaction

To determine if disulfide bridge can be replaced by salt bridge or hydrophobic interaction, Cys residues were substituted by negatively and positively charged residues or hydrophobic residues (confirmed by MS analysis as referred in **Table S3**). Bovicin HJ50 mutant C21D/C29K (D/K) was inactive against *M. flavus* NCIB8166 even when applied at 40 µg/mL (**Figure 5D**), indicating that salt bridge could not substitute disulfide bridge in bovicin HJ50 in restoring its antimicrobial activity. D/K mutation in suicin, cerecin and perecin also abolished their antimicrobial activity (**Figure 5D**). When it came to hydrophobic interactions, bovicin HJ50 mutant C21L/C29L (L/L) and C21F/C29F (F/F) showed no antimicrobial activity (**Figure 5D**), indicating the irreplaceable role of disulfide bridge by hydrophobic interaction. L/L and F/F substitution in cerecin and perecin showed the same results (**Figure 5D**). However, while both single point mutants (C21L or C21F) of suicin were inactive (data not shown), L/L and F/F mutants retained reduced activity at high concentration (20 µg/mL), indicating that hydrophobic interaction could retain attenuated activity of suicin. Thus, it could be concluded that neither electrostatic nor hydrophobic interaction could substitute disulfide bridge in most bovicin HJ50-like lantibiotics.

### Disulfide breakage impairs the ability to compromise cell membrane

**Figure 6:**
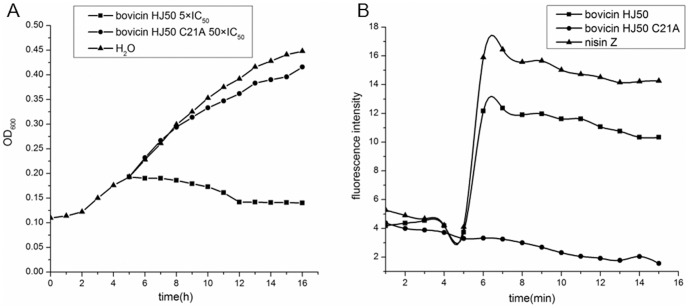
Inhibition of bacterial growth and disruption of membrane by bovicin HJ50. (A) Influence of bovicin HJ50 and its mutant C21A on bacterial growth. The growth curve of *M. flavus* NCIB8166 was recorded when bovicin HJ50 of 5×IC_50_ (square dotted curve), its mutant C21A of 50×IC_50_ (circle dotted curve) and H_2_O (triangle dotted curve) was added at the time point of 5 h. (B) Influence of membrane potential when bovicin HJ50 and its mutant C21A were added to *M. flavus* NCIB8166. The fluorescence intensity was monitored in a time duration of 15 min. bovicin HJ50 (square dotted curve) and its mutant C21A (circle dotted curve) was added at the time point of 5 min. Nisin Z (triangle dotted curve) was used as positive control.

Bacterial growth inhibitory assay showed that, bovicin HJ50 of lethal concentration (5× IC_50_) was bacteriocidal to *M. flavus* NCIB8166 whereas its C21A mutant exhibited no activity even with the 50-fold concentration of IC_50_ (**Figure 6A**). As membrane disruption is an important mechanism of bactericidal action of lantibiotics as evidenced by nisin and suicin [17], [19], membrane potential changes in *M. flavus* NCIB8166 were monitored by DiBAC4(3) (bis-(1,3-dibutylbarbituric acid) trimethine oxonol) when bovicin HJ50 or its C21A mutant was applied to the indicator strain. As a result, the increase of fluorescence intensity of DiBAC4(3) after addition of bovicin HJ50 indicated that bovicin HJ50 disrupted the membrane potential of *M. flavus* NCIB8166 (**Figure 6B**). Whereas in the case of its C21A mutant, the fluorescence intensity was not substantially changed (**Figure 6B**). This further indicated that disulfide bridge was crucial for bovicin HJ50 to disrupt the membrane of the target cells.

### Impact of disulfide breakage on secondary structure

**Figure 7:**
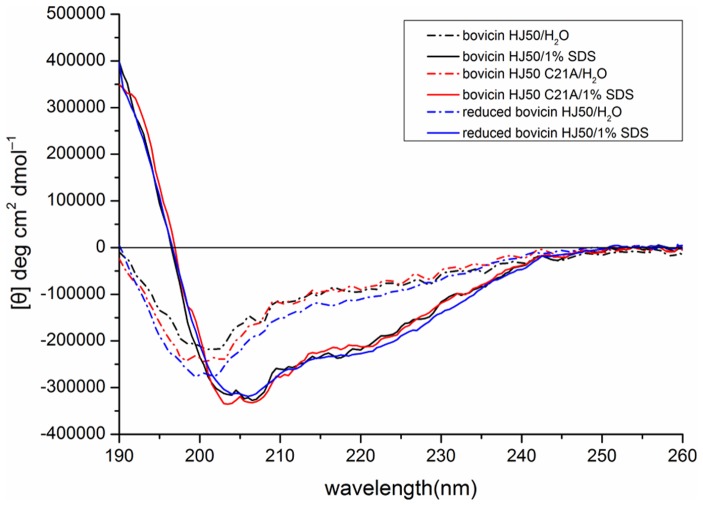
Circular dichroism (CD) analysis of bovicin HJ50 and its mutants. Dash-dot-dash line represented the secondary structures of bovicin HJ50 (black), its mutant C21A (red) and reduced form in H_2_O, while solid line represented the secondary their structures in 1% SDS.

To determine the structural contribution of disulfide bridge, secondary structure of wild-type, reduced and C21A mutant of bovicin HJ50 were determined by circular dichroism (CD). As shown in **Figure 7**, bovicin HJ50 adopted an unordered structure in H_2_O whereas was induced into a helix-prone structure in 1% SDS of membrane mimicking environment. As measured by CDNN software, the helical propensity of bovicin HJ50 was increased when assayed in 1% SDS condition (data not shown). Interestingly, similar structure was also observed in reduced form and C21A mutant of bovicin HJ50 C21A in either H_2_O or 1% SDS (**Figure 7**). The helical propensity of C21A in H_2_O was lower than that in SDS condition. This indicated that disulfide bridge was not contributive to the secondary structure which might be ascribed to the constrained structure mainly maintained by thioether bridges (ring A and B) of bovicin HJ50. Although the N-terminal linear part of bovicin HJ50 was shorter compared with lacticin 481-like lantibiotics, it was predicted to be an α helix-prone fragment. Thus, the disulfide bridge was not influential to the secondary structure of bovicin HJ50.

### Impact of disulfide bridge broken on hydrophobicity

**Figure 8:**
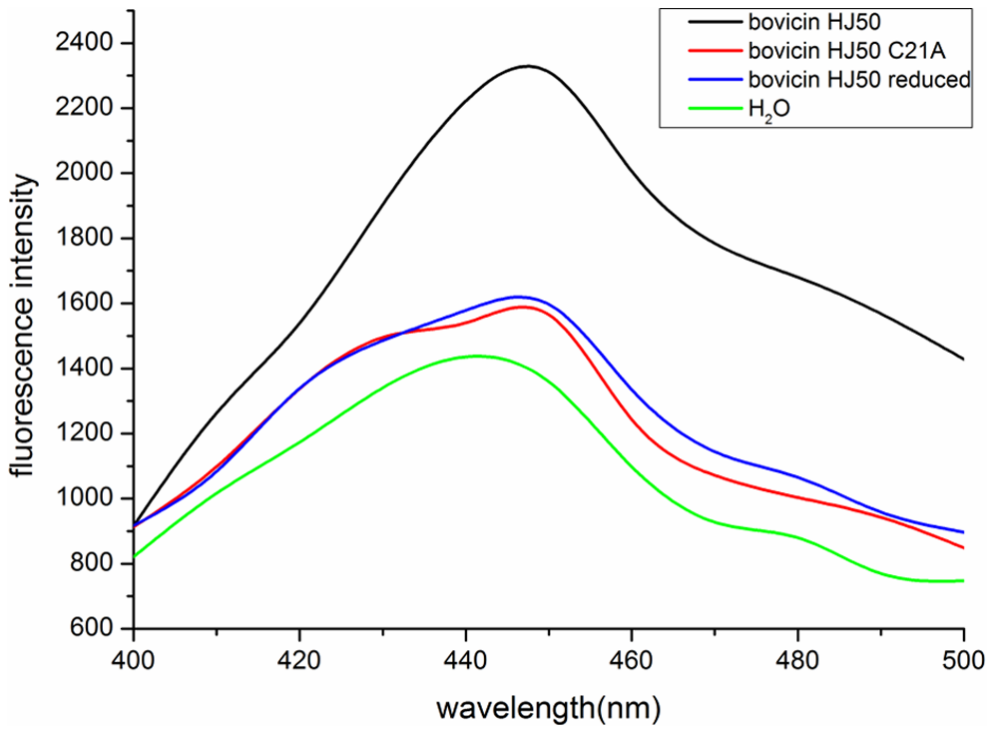
Comparison of hydrophobicity of bovicin HJ50 and its mutants by ANS binding analysis. Bovicin HJ50 (black curve), its reduced form (blue) and mutant C21A (red). H_2_O was used as negative control (green).

Hydrophobicity prediction by Protscale (http://web.expasy.org/protscale/) [20] envisaged that ring C, maintained by disulfide bridge, was the hydrophobic core region of bovicin HJ50-like lantibiotics (**Figure S4**). The contribution of disulfide bridge to hydrophobicity of bovicin HJ50 was determined by C_18_ RP HPLC and ANS (bis-(8-anilinonaphthalene-1-sulfonate)) binding analysis. RP HPLC showed that bovicin HJ50 was elucidated at 17.5 min, reduced bovicin HJ50 at 15.7 min (**Figure 4B**) and C21A mutant at 15.6 min (data not shown). This suggested that breakage of disulfide bridge lowered the hydrophobicity of bovicin HJ50. To support that, ANS binding assay was applied to assess the surface hydrophobicity of wild-type, reduced and C21A mutant of bovicin HJ50. The decrease of fluorescence strength of reduced and C21A mutant of bovicin HJ50 indicated lower hydrophobicity compared to wild type (**Figure 8**), implying that disulfide bridge could maintain the hydrophobicity of bovicin HJ50.

Prompted by the fact that the hydrophobicity of ring C maintained by disulfide bridge was critical to the efficacy of bovicin HJ50, Val substitution mutagenesis was conducted in ring C. Except T24V, whose activity was slightly decreased, other mutants including A22V, T27V and A28V exhibited higher potency than wild type bovicin HJ50, which was reflected by lower IC_50_ value in inhibiting *M. flavus* NCIB8166 (**Table 2**). This underpinned the crucial role of hydrophpbicity of ring C in the function of bovicin HJ50. Substitution of ring C with the counterpart of suicin, cerecin and perecin restored full modification including the disulfide bridge formation (**Table 2**). These chimeric mutants exhibited comparable activity with respective wild type lantibiotics, further underlining the crucial role of ring C in activity of these lantibiotics. However, by using two residues (Pro and Gly) to substitute the ring C of bovicin HJ50 or suicin, the modification was fully retained but the activity was totally abolished (**Table 2**).

**Table 2:** MS analysis and IC_50_ determination of ring C mutants of bovicin HJ50.

Mutants	MW cal. (Da)	MW by MS (Da)	ΔMW (Da)	PTM	IC_50_ (µg/mL)
A22V	3494.76	3456.65	38.11	2H_2_O+S-S	0.25
T24V	3464.75	3426.65	38.10	2H_2_O+S-S	1
T27V	3464.75	3426.66	38.09	2H_2_O+S-S	0.125
A28V	3494.76	3456.65	38.11	2H_2_O+S-S	0.125
C-s^a^	3626.79	3588.61	38.18	2H_2_O+S-S	0.2
C-p	3777.8	3739.67	38.13	2H_2_O+S-S	2
C-c	3719.92	3681.80	38.12	2H_2_O+S-S	1
C-PG	2993.45	2955.34	38.11	2H_2_O+S-S	-b

a C-s, C-p, C-c and C-PG respectively represents the mutant of substitution of c ring of bovicin HJ50 by the counterpart of suicin, perecin, cerecin and ProGly.

b No antimicrobial activity detected.

## Discussion

Lantibiotics are gene-encoded peptide antibiotics with remarkable effectiveness against Gram-positive pathogens. Because of their special mode of action without raising substantial resistance, lantibiotics implies great potential to be attractive options for food and medical uses. Thus, discovery of new lantibiotics are appealing and urgent. Recently, genomic mining has greatly advanced the discovery of novel lantibiotic gene clusters, especially accompanying with roaring expansion of sequenced microbial genomes [21]–[23]. Bovicin HJ50 was firstly recognized as a unique class II lantibiotic produced by *S. bovis* HJ50 containing an unusual disulfide bridge. Later, by searching analogs of BovA in NCBI database, an amazing quantity of BovA-identical or -like precursor peptides and corresponding *lan* loci were revealed in a wide variety of bacteria, including various pathogenic strains. Following PCR screening of BovA-like precursors, three representative *lan* loci designated *per*, *bce* and *thu* were unveiled in this study in *C. perfringens*, *B. cereus* and *B. thuringiensis*, respectively. These lantibiotic gene clusters were similar with *bov* locus in organization and the biosynthesis-related proteins showed remarkable identities, indicating the same function to produce lantibiotics. However, all the three strains were not reported or found to produce any lantibiotics. Hence, the counterpart mature and bioactive lantibiotics named perecin, cerecin and thuricin were generated via SIVB [14]. Including previously synthesized suicin [17], these lantibiotics were supposed to be categorized into a novel subgroup designated bovicin HJ50-like lantibiotics, which are characterized by containing a typical disulfide bridge.

Structural elucidation indicated that bovicin HJ50-like lantibiotics were different from lacticin 481-like lantibiotics. Though both of them are conferred with an N-terminal linear and C-terminal globular structure, the ring C at C terminus is maintained by a disulfide bridge in bovicin HJ50-like lantibiotics but by a thioether bridge in lacticin 481-like lantibiotics (**Figure 3A**). Attempts to replace the disulfide bridge of bovicin HJ50 with thioether bridge by substitution of Cys21 with either Ser or Thr were unsuccessful (data not shown), though flanking residues of Cys21 were hydrophobic that would facilitate dehydration [24]. Furthermore, the catalytic promiscuity of BovM to yield suicin with the leader peptide of either BovA or SuiA suggested that bovicin HJ50-like lantibiotics are grouped into the same category as found in lacticin 481-like lantibiotics [17], [25]. Moreover, although the mode of action of bovicin HJ50-like lantibiotics remains enigmatic, it seemed to be different from that of lacticin 481. The ring A, which was almost identical in bovicin HJ50-like lantibiotics and conserved in lacticin 481-like lantibiotics, has been recognized as a lipid-II binding motif [26], [27]. This implied that bovicin HJ50-like lantibiotics might exert function by targeting lipid-II as lacticin 481. However, unlike lacticin 481 lacking pore-forming capacity on the membrane of susceptible bacteria [27], bovicin HJ50-like lantibiotics such as bovicin HJ50 and suicin are capable of forming pores on cell membrane, which leads to leakage of ions and collapse of membrane potential [13], [17]. Collectively, bovicin HJ50 subgroup differ from lacticin 481 subgroup not only in structure but also in function.

Although the disulfide bridge is conserved among bovicin HJ50-like lantibiotics, no specific enzyme was identified to be responsible for disulfide bridge formation in the producing bacteria. Downstream of *bovI*, a *dsbA* gene encoding a thiol/disulfide oxidoreductase was not responsible for disulfide formation, which is consistent with the fact that no *dsbA* genes were found in *sui*, *per*, *bce* and *thu* loci [28]. BovM was firstly suggested to be involved in introduction of disulfide bridge in bovicin HJ50 at the same time of introducing thioether bridge. However, *in vitro* modification of BovM towards BovA was hindered in the absence of reductive reagents because of the randomly cross-linked disulfide connections as also observed in lacticin 481 [29]. This suggested the reducing conditions were prerequisite for the function of BovM. Actually, in reducing conditions, BovM produced correctly modified bovicin HJ50 without disulfide bridge, indicating its irrelevance to disulfide bridge formation. In contrast, it was proved here that the disulfide bridge was not enzymatically catalyzed but spontaneously formed. Intriguingly, bovicin HJ50 contains a putative γ-core motif CXG (Cys21/Ala22/Gly23) in ring C that has been found in disulfide-containing antimicrobial peptides produced by organisms phylogenetically distant from bovicin HJ50-producing bacteria [30]. Thus, it was suggested that disulfide bridge formed spontaneously, which might be dependent on the ring C constituents.

Herein, the disulfide bridge was substantiated to be indispensable for the full antimicrobial function of bovicin HJ50-like lantibiotics. Breakage of disulfide bridge by Ala mutation or alkylation by NEM abolished their bioactivity, which was reinforced by the fact that bovicin HJ50 C21A mutant was unable to disrupt membrane potential of indicator strain *M. flavus* NCIB8166 (**Figure 5** and **6**). In contrast to the conserved disulfide bridge of IIa bacteriocins like leucocin A [31], the disulfide bridge in bovicin HJ50-like lantibiotics was intolerant of substitution by non-covalent bond such as hydrophobic and electrostatic interaction (**Figure 5**). However, the antimicrobial activity of reduced bovicin HJ50 seemed ambiguous in that bovicin HJ50 retained decreased activity while the identical thermophilin 1277 abolished activity when treated with reductive reagents [15]. It was postulated that the reduced disulfide bridge could be spontaneously re-formed in oxidative conditions to exhibit activity. This raised an intriguing hypothesis that the disulfide bridge might function as a redox switch to control the efficacy of bovicin HJ50-like lantibiotics in a redox-regulation mechanism as human β-defensin 1 [32].

However, the question was raised as to how disulfide bridge in bovicin HJ50-like lantibiotics supports their bacteriocidal functions. CD analysis of wild type, reduced and C21A mutant of bovicin HJ50 excluded the contribution of disulfide bridge to the structure of bovicin HJ50 as all versions of the lantibiotic had the same secondary structure in either H_2_O or membrane mimicking conditions (**Figure 7**). On the contrary, disulfide bridge seems to be contributing to hydrophobicity of bovicin HJ50 in that reduced or C21A mutant of the lantibiotic displayed decreased retention time in RP-HPLC (**Figure 4**). This was further supported by ANS binding experiments that wild type bovicin HJ50 could bind more ANS than its reduced version or C21A mutant. However, increasing its hydrophobicity by replacing residues near disulfide bridge within ring C with Val enhanced bacteriocidal activity of bovicin HJ50. Interestingly, although the ring C among bovicin HJ50-like lantibiotics showed little sequence homology, substitution of ring C of bovicin HJ50 with that of suicin, perecin or cerecin did not interfere the activity of bovicin HJ50. It was thus proposed that disulfide bridge evolved specifically just as the identical lipid II-binding ring A to retain the function of bovicin HJ50-like lantibiotics, while the specific constituent of ring C greatly determined the efficacy of each lantibiotic.

In conclusion, from a series of untapped lantibiotic loci revealed by genomic mining, three representative bovicin HJ50-like lantibiotics were biosynthesized and characterized. In this special disulfide-containing lantibiotic subgroup, disulfide bridge was substantiated to be crucial for their antimicrobial activity probably by maintaining the hydrophobicity of bovicin HJ50-like lantibiotics. Besides involvement in bacteriocidal function of bovicin HJ50-like lantibiotics, disulfide bridge in bovicin HJ50 was recently shown to play a critical role in auto-inducing function of bovicin HJ50 to trigger the two component system BovK/BovR [33]. Thus, the prevalence of disulfide-containing bovicin HJ50-like lantibiotics provided new structural diversity to lantibiotics, which might also promote new understanding of the function of disulfide bridge in lantibiotics and spur consideration of the roles of these disulfide-containing lantibiotics in separate niches.

## Materials and Methods

### Bacterial strains and growth conditions

*Escherichia coli* DH5α was used as host for DNA cloning and BL21(DE3) for protein expression. The *Bacillus cereus* strains (As 1.1846, 1.260, 1.252, 1.348 and 1.447) and *B. thuringiensis* strains (As 1.913, 1.1013 and 1.1014) were obtained from China General Microbiological Culture Collection Centre (CGMCC, Beijing, China). The genomic DNA of *Clostridium perfringens* D str. JGS1721 was provided by Dr. J. Glenn Songer from Iowa State University. The *E. coli* and bacilli strains were incubated in Luria-Bertani (LB) broth (tryptone (1%), yeast extract (0.5%), sodium chloride (1%)) at 37°C and kanamycin (50 µg/mL) was added if necessary. *Micrococcus flavus* NCIB8166 was inoculated in S1 medium (tryptone (0.8%), yeast extract (0.5%), glucose (0.5%), disodium hydrogen phosphate (0.2%), sodium chloride (0.5%), Tween-20 (0.1%)) at 30°C. TCEP (tris(2-carboxyethyl) phosphine), DTT (DL-Dithiothreitol), NEM (N-ethylmaleimide), DiBAC4(3) (bis-(1,3-dibutylbarbituric acid) trimethine oxonol) and ANS (bis-(8-anilinonaphthalene-1-sulfonate)) were purchased from Sigma-Aldrich company.

### Molecular cloning protocols

Molecular biology techniques were carried out according to standard protocols [34]. PCR was performed using TransStart *FastPfu* DNA polymerase (Transgene, China) according to the manufacturer's protocol. Plasmids, PCR products or DNA fragments were prepared or purified by Axygen kits according to respective instructions (Axygen, USA). Genomic DNA of bacilli was extracted and purified using MOPS method as described in [35]. Probe primers were designed as follows: forward, ATGGAAACTGAAAAATATTTA; reverse, GCATTTTTTACAAAT. PCR screening was performed towards bacilli strains with probe primers. Once positive strains detected, the complete lantibiotic gene clusters were amplified and ligated to pEASY-Blunt cloning plasmids (Transgene, China) for sequencing analysis. *bceA* was amplified with primers containing *Nde*I and *BamH*I and *bceM* with primers containing *Bam*HI and *Xho*I. They were successively constructed into pET28a with *Bam*HI in between to obtain pET28a-*bceAM*. *thuAM* and *perAM* was amplified with primers containing *Nde*I and *Xho*I and constructed to obtain pET28a-*thuAM* and pET28a-*perAM*. *bovA* and *bovM* were amplified from genomic DNA of *S. bovis* HJ50 and constructed into pET28a to obtain pET-*bovA* and pET-*bovM*. Site-specific ligase-independent mutagenesis (SLIM) was performed via a PCR method with primers containing corresponding mutations as referred to the protocols described by *Chiu* [36].

### Protein expression and purification

*E. coli* BL21(DE3) transformed with expression vectors were incubated in LB broth (1 L) at 37°C. Isopropyl β-D-1-thiogalactopyranoside (IPTG) was added to a final concentration of 0.5 mM when the optical density at 600 nm (OD_600_) of cell culture reached 0.6–0.7 and the cells were then induced for another 20 h at 16°C. Induced cells were harvested by centrifugation at 5,000×g for 20 min and resuspended in 30 ml binding buffer (25 mM Na_2_HPO_4_, 25 mM NaH_2_PO_4_, 500 mM NaCl, pH 7.4) plus 20 mM imidazole. After cells were ruptured by sonication, centrifugation was performed at 10,000×g for 30 min to separate supernatant and inclusion bodies. The supernatant was applied to immobilized metal affinity chromatography (IMAC) with Ni^2+^ column in a stepwise wash with binding buffer plus 40 mM imidazole and finally the target protein was eluted with binding buffer plus 500 mM imidazole. The inclusion bodies were dissolved in binding buffer containing 8 M urea and purified by IMAC as described previously [17].

### *In vitro* cleavage of leader peptide

His_6_-BovT_150_ was expressed as described by *Lin* [14] and applied here to remove the leader peptides of bovicin HJ50-like lantibiotics. Purified modified precursor peptides were incubated with His_6_-BovT_150_ at 25°C for 4 h. The reaction buffer (pH 7.4) contained 50 mM Na_2_HPO_4_, 50 mM Na_2_SO_4_ and 1 mM DTT. Then His_6_-BovT_150_ was removed by heating at 60°C for 10 min and subsequent centrifugation at 12,000×g for 10 min. Reverse phase high performance liquid chromatograph (RP HPLC) was performed with a C_18_ analysis column (Shimadzu, Japan) using a water-acetonitrile solvent system. The standard HPLC method was following the instructions as described previously [17]. Purified protein were determined by Tricine-SDS-PAGE as described in [37] and protein concentration was quantified by standard BCA assay kit (Thermo Scientific, USA).

### *In vitro* assay of BovM

His_6_-BovM was purified to determine its *in vitro* modification function towards His_6_-BovA. The reaction buffer contained 50 mM Tris, 10 mM MgCl_2_, 2.5 mM ATP, 1 mM DTT (pH 7.4). His_6_-BovA was incubated with His_6_-BovM in the reaction buffer at 25°C for 1 h. After modification, His_6_-BovA was incubated with His_6_-BovT_150_ to remove the leader peptide.

### Antimicrobial assay and 50% inhibitory concentration (IC_50_) determination

Antimicrobial activity was assayed against sensitive indicator strain *M. flavus* NCIB8166 with agar diffusion method. *M. flavus* NCIB8166 was grown in liquid S1 medium at 30°C overnight and then was transferred to molten solid S1 medium (1.5% agar) to prepare plate. Antimicrobials of 25 µL were applied to holes with diameter of 5 mm on the plates. The 50% inhibitory concentration (IC_50_) was defined as the lowest concentration of antimicrobials that could cause inhibition of visible growth of indicator strains by 50% compared to the initial OD_600_. It was determined against sensitive strain *M. flavus* NCIB 8166 with micro-dilution method with 96-well plate as described by *Levengood* [38]. Bacterial growth inhibitory assay was performed by applying antimicrobial compounds to the indicator strain *M. flavus* NCIB 8166 and recording the OD_600_ at time interval of 1 h.

### Chemical modification and MS analysis

Bovicin HJ50 and its analogues were subjected to NEM (N-ethylmaleimide) for chelating free thiols, resulting in 125 Da increase in mass per thiol. NEM reaction was performed by incubation of purified sample with 1 mM TCEP and 10 mM NEM (pH 7.5) in ice bath for 30 min. After reaction, the samples were applied to C_18_ Zip-tip and detected by matrix-assisted laser desorption ionization-time of flight mass spectrometry (MALDI-TOF MS). MALDI-TOF MS analysis was performed on 4700 Proteomics Analyzer mass spectrometer (Applied Biosystems) to identify purified peptides. Mass spectra were obtained in the positive reflectron mode for peptides lower than 5 kDa and linear mode for peptides larger than 5 kDa. α-cyano-4-hydroxycinnamic acid (CHCA) matrix was prepared by dissolving 5 mg in 1 ml of 50∶50 acetonitrile/water containing 0.1% trifluoroacetic acid (TFA).

### Circular dichroism analysis

Circular dichroism (CD) spectroscopy of the purified peptides were determined with a Jasco J-810 circular dichroism spectropolarimeter (Applied Photophysics) at 25°C in sterilized water or membrane mimicry circumstance of 1% SDS [39]. Lyophilized peptides were added in above mentioned solutions to a final concentration of 120 µg/mL and were loaded into a Quartz cell of 0.1 cm. The molar ellipticity was scanned from 190 to 260 nm with a spectral bandwidth of 1 nm. Analysis of the secondary structure was performed with Pro-Data and CDNN software. All spectra were background subtracted and the data were normalized to molar ellipticity.

### ANS binding analysis

ANS is a hydrophobic dye whose fluorescence increases upon binding to hydrophobic sites and has been widely used to assess the surface hydrophobicity of proteins [32]. Peptide samples were diluted to a concentration of 50 µg/mL with sodium acetate buffer (50 mM, pH 4.0) containing 10 µM ANS. After 1 h of incubation in the dark, fluorescence spectra were monitored by microplate reader (BioTek) at the excitation wavelength of 375 nm and the emission wavelengths ranging from 400 to 500 nm were recorded.

### Membrane disruption determination

Membrane potential disruption was monitored by DiBAC4(3), which is a sensitive membrane potential probe that measures potential-dependent changes by accompanied fluorescence changes [40]. The membrane potential changes were determined against indicator strain *M. flavus* NCIB8166 when subjected respectively to bovicin HJ50 (5×IC_50_) and its mutant C21A (50×IC_50_). The instructions were referred as described previously [17]. Nisin Z (5×IC_50_) was used as positive control.

## Supporting Information

Figure S1 **Sequence alignment of BovA-like peptides.**
(TIF)

Figure S2 **MS analysis of bovicin HJ50-like lantibiotics.** MS analysis of suicin (A), perecin (B), cerecin (C) and thuricin (D).
(TIF)

Figure S3 **MS/MS analysis of bovicin HJ50 produced by BovM in the absence of DTT.**
(TIF)

Figure S4 **Hydrophobicity profile prediction of bovicin HJ50-like lantibiotics.** The black line stands for bovicin HJ50, red line for suicin, blue line for perecin, pink line for cerecin and green line for thuricin.
(TIF)

Table S1 **BovA-like lantibiotic precursor peptides in NCBI database.**
(DOCX)

Table S2 **MS analysis of ring disruption mutants of perecin and cerecin.**
(DOCX)

Table S3 **MS analysis of disulfide substitution mutants of bovicin HJ50-like lantibiotics.**
(DOCX)
