# Supplementary figures and images for: Bovicin HJ50-Like Lantibiotics, a Novel Subgroup of Lantibiotics Featured by an Indispensable Disulfide Bridge
Source: PLoS One. 2014 May 12;9(5):e97121. doi: 10.1371/journal.pone.0097121 (PMC4018250; doi:10.1371/journal.pone.0097121)

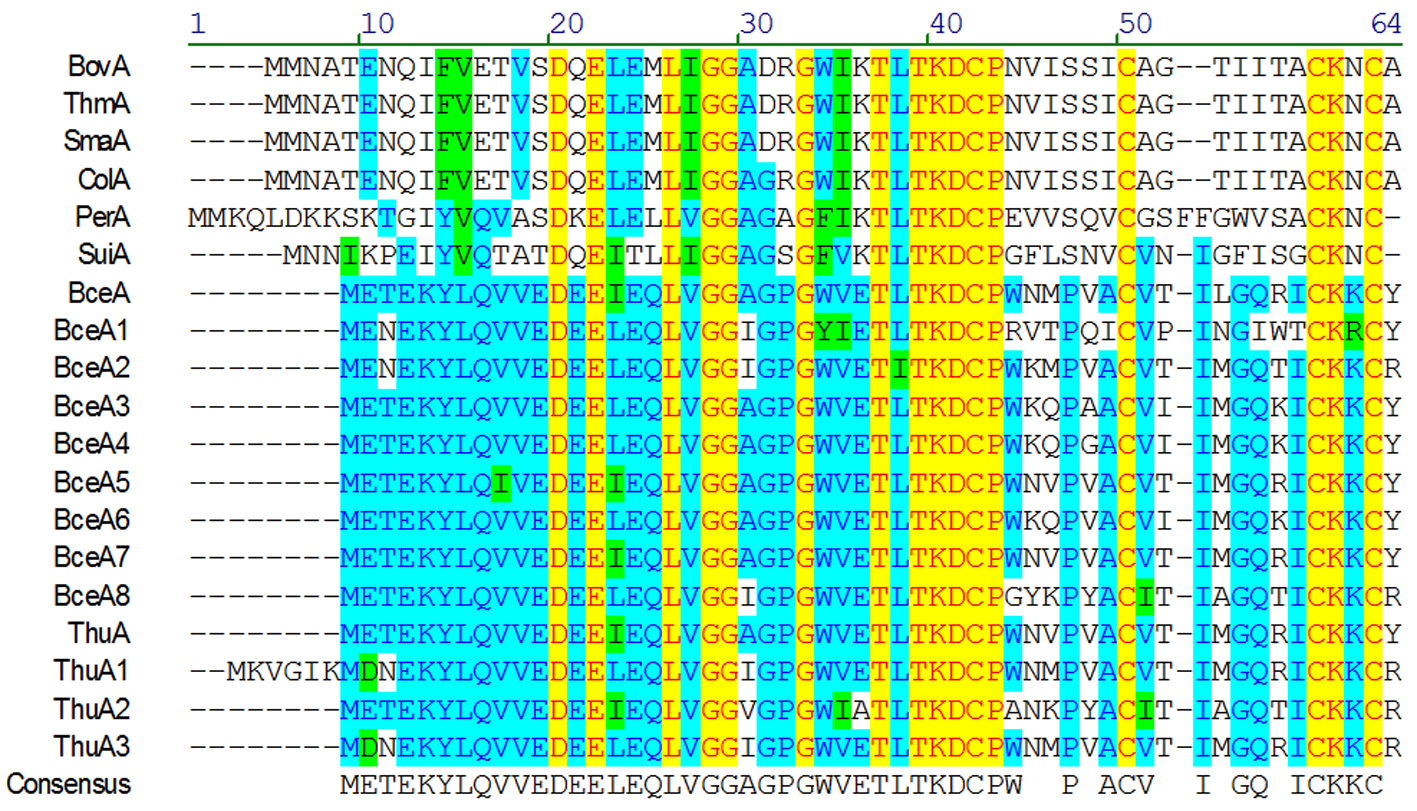

Supplement: Figure S1 — Sequence alignment of BovA-like peptides. (TIF) [file pone.0097121.s001.tif]

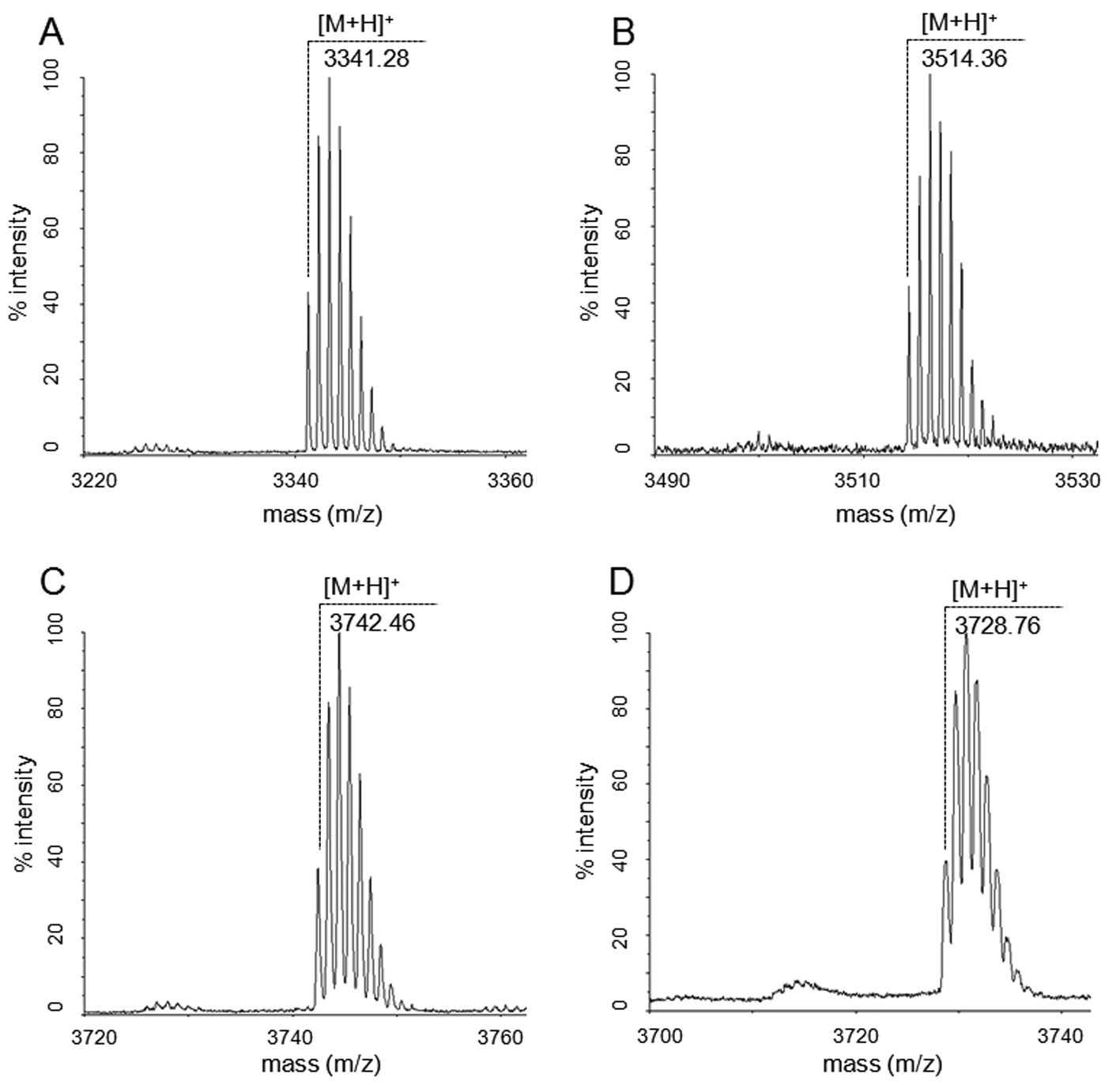

Supplement: Figure S2 — MS analysis of bovicin HJ50-like lantibiotics. MS analysis of suicin (A), perecin (B), cerecin (C) and thuricin (D). (TIF) [file pone.0097121.s002.tif]

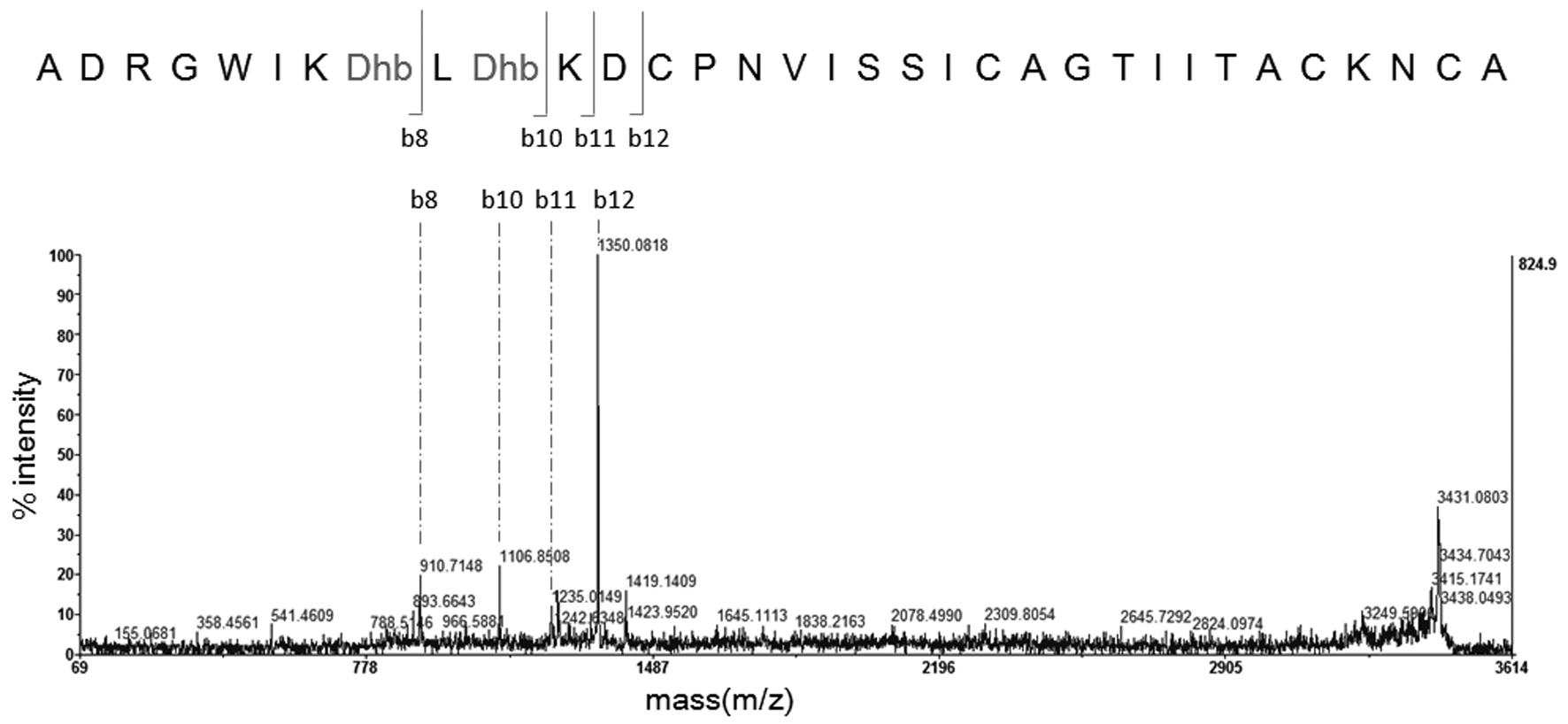

Supplement: Figure S3 — MS/MS analysis of bovicin HJ50 produced by BovM in the absence of DTT. (TIF) [file pone.0097121.s003.tif]

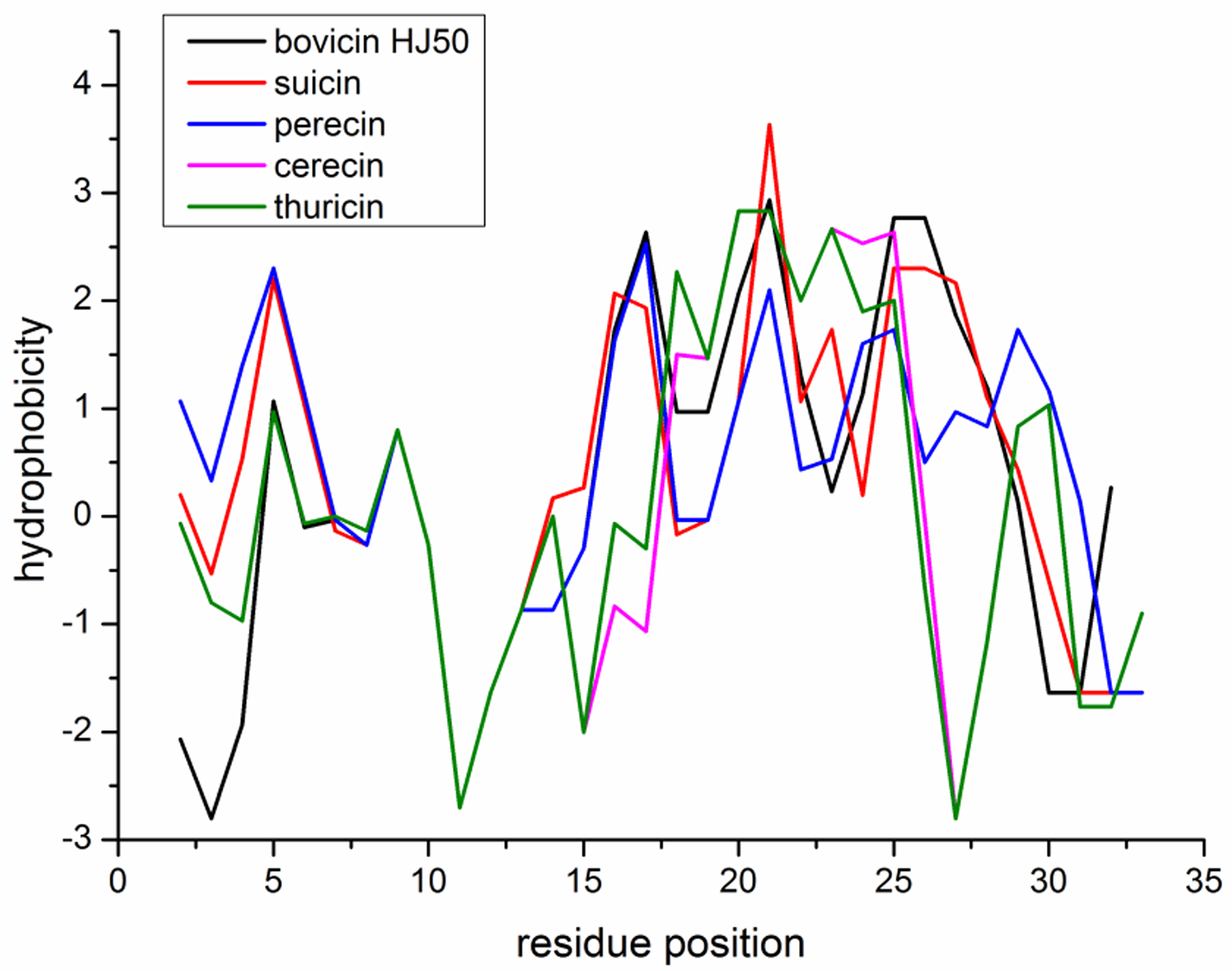

Supplement: Figure S4 — Hydrophobicity profile prediction of bovicin HJ50-like lantibiotics. The black line stands for bovicin HJ50, red line for suicin, blue line for perecin, pink line for cerecin and green line for thuricin. (TIF) [file pone.0097121.s004.tif]
