# Supplementary material for: Bovicin HJ50-Like Lantibiotics, a Novel Subgroup of Lantibiotics Featured by an Indispensable Disulfide Bridge
Source: PLoS One. 2014 May 12;9(5):e97121. doi: 10.1371/journal.pone.0097121 (PMC4018250; doi:10.1371/journal.pone.0097121)
Supplement: Table S1 — BovA-like lantibiotic precursor peptides in NCBI database. (DOCX) [file pone.0097121.s005.docx]

**Table S1.** BovA-like lantibiotic precursor peptides in NCBI database.

| **Prepeptide** | **Origination** | **Accession no.** | **Identity with BovA(%)** |
| --- | --- | --- | --- |
| BovA | *Streptococcus bovis* HJ50 | AAP23217 | 100 |
| ThmA | *Streptococcus thermophilus* SBT1277 | BAF75720 | 100 |
|  | *Streptococcus thermophilus* TH1477 | EWM62233 |  |
| SmaA | *Streptococcus macedonicus* ACA-DC 198 | CCF02700 | 100 |
| ColA | *Enterococcus columbae* PLCH2 | ABJ98063 | 100 |
| SuiA | *Streptococcus suis* SC84 | YP_003024876 | 49 |
|  | *Streptococcus suis* 05ZYH33 | -*^a^* |  |
|  | *Streptococcus suis* 98HAH33 | - |  |
|  | *Streptococcus suis* D12 | YP_006082846 |  |
| PerA | *Clostridium perfringens* D str. JGS1721 | EDT72694 | 45 |
| BceA1 | *Bacillus cereus* 172560W | EEK58956 | 44 |
|  | *Bacillus cereus* BAG3X2-2 | EJQ15572 |  |
|  | *Bacillus cereus* VD045 | EJR29440 |  |
|  | *Bacillus cereus* VD156 | EJR73190 |  |
|  | *Bacillus cereus* str. Schrouff | EOO05760 |  |
|  | *Bacillus cereus* K-5975c | EOO81772 |  |
| BceA2 | *Bacillus cereus* F65185 | EEL64077 | 39 |
| BceA3 | *Bacillus cereus* Rock3-29 | EEL36812 | 39 |
| BceA4 | *Bacillus cereus* BAG4X2-1 | EJV41820 | 39 |
|  | *Bacillus cereus* BAG6O-1 | EJV42216 |  |
|  | *Bacillus cereus* HuB2-9 | EJV89750 |  |
|  | *Bacillus cereus* HuA2-3 | EOP32214 |  |
|  | *Bacillus cereus* VD214 | EOP47105 |  |
| BceA5 | *Bacillus cereus* VD048 | EJR27159 | 37 |
| BceA6 | Bacillus cereus VD021 | EOO65286 | 39 |
| BceA7 | *Bacillus cereus* HuB13-1 | EOP30914 | 37 |
|  | *Bacillus cereus* ISP2954 | EOP62663 |  |
|  | *Bacillus cereus* BMG1.7 | EOP88709 |  |
| BceA8 | *Bacillus cereus* BAG1X1-1 | EOO25092 | 39 |
|  | *Bacillus cereus* BAG1X2-1 | EOO44017 |  |
|  | *Bacillus cereus* BAG1X2-2 | EOO46159 |  |
|  | *Bacillus cereus* BAG1X2-3 | EOO62606 |  |
|  | *Bacillus cereus* BAG2O-1 | EOP01588 |  |
| ThuA1 | *Bacillus thuringiensis* ATCC 10792 | EEM62479 | 36 |
|  | *Bacillus thuringiensis* T01001 | EEM31334 |  |
| ThuA2 | *Bacillus thuringiensis* IBL 200 | EEM95366 | 39 |
| ThuA3 | *Bacillus thuringiensis* IS5056 | AGG04873 | 37 |

*^a^* Not annotated in the genome in NCBI database but re-annotated in ref. [17].
