# Supplementary material for: Bovicin HJ50-Like Lantibiotics, a Novel Subgroup of Lantibiotics Featured by an Indispensable Disulfide Bridge
Source: PLoS One. 2014 May 12;9(5):e97121. doi: 10.1371/journal.pone.0097121 (PMC4018250; doi:10.1371/journal.pone.0097121)
Supplement: Table S2 — MS analysis of ring disruption mutants of perecin and cerecin. (DOCX) [file pone.0097121.s006.docx]

**Table S2.** MS analysis of ring disruption mutants of perecin and cerecin.

| **lantibiotics** | **Mutants** | **MW cal. (Da)** | **MW by MS (Da)** | **ΔMW(Da)** | **PTM** |
| --- | --- | --- | --- | --- | --- |
| perecin | C13A | 3520.71 | 3482.62 | 38.09 | 2H_2_O+1S-S |
|  | C21A | 3520.71 | 3484.73 | 35.98 | 2H_2_O |
|  | C31A | 3520.71 | 3484.74 | 35.97 | 2H_2_O |
|  | C34A | 3520.71 | 3482.59 | 38.12 | 2H_2_O+1S-S |
| cerecin | C13A | 3748.88 | 3710.71 | 38.17 | 2H_2_O+1S-S |
|  | C21A | 3748.88 | 3712.52 | 36.36 | 2H_2_O |
|  | C30A | 3748.88 | 3712.53 | 36.35 | 2H_2_O |
|  | C33A | 3748.88 | 3710.73 | 38.15 | 2H_2_O+1S-S |
