# Supplementary material for: Bovicin HJ50-Like Lantibiotics, a Novel Subgroup of Lantibiotics Featured by an Indispensable Disulfide Bridge
Source: PLoS One. 2014 May 12;9(5):e97121. doi: 10.1371/journal.pone.0097121 (PMC4018250; doi:10.1371/journal.pone.0097121)
Supplement: Table S3 — MS analysis of disulfide substitution mutants of bovicin HJ50-like lantibiotics. (DOCX) [file pone.0097121.s007.docx]

**Table S3.** MS analysis of disulfide substitution mutants of bovicin HJ50-like lantibiotics.

| **Lantibiotics** | **Mutants** | **MW cal. (Da)** | **MW by MS (Da)** | **ΔMW(Da)** | **PTM** |
| --- | --- | --- | --- | --- | --- |
| bovicin HJ50 | C21D/C29K | 3503.83 | 3467.61 | 36.22 | 2H_2_O |
|  | C21L/C29L | 3486.88 | 3450.64 | 36.24 | 2H_2_O |
|  | C21F/C29F | 3554.85 | 3518.62 | 36.23 | 2H_2_O |
| suicin | C21D/C30K | 3416.73 | 3380.66 | 36.07 | 2H_2_O |
|  | C21L/C30L | 3399.78 | 3363.62 | 36.16 | 2H_2_O |
|  | C21F/C30F | 3467.75 | 3431.56 | 36.19 | 2H_2_O |
| perecin | C21D/C31K | 3589.78 | 3553.55 | 36.23 | 2H_2_O |
|  | C21L/C31L | 3572.83 | 3536.65 | 36.18 | 2H_2_O |
|  | C21F/C31F | 3640.79 | 3604.64 | 36.15 | 2H_2_O |
| cerecin | C21D/C30K | 3817.96 | 3781.79 | 36.17 | 2H_2_O |
|  | C21L/C30L | 3801.01 | 3764.76 | 36.25 | 2H_2_O |
|  | C21F/C30F | 3868.97 | 3832.73 | 36.24 | 2H_2_O |
